# Impact of nutrient density diet with emulsifier supplementation on apparent total tract digestibility and ileal amino acid digestibility in broilers

**DOI:** 10.1016/j.psj.2024.104593

**Published:** 2024-11-26

**Authors:** Shanmugam Suresh Kumar, Jae Hong Park, In Ho Kim

**Affiliations:** a Department of Animal Biotechnology, Dankook University, Cheonan, 31116, Republic of Korea; b Smart Animal Bio Institute, Dankook University, Cheonan, Republic of Korea

**Keywords:** Broilers, Emulsifier, Apparent total tract digestibility, Apparent ileal digestibility, Amino acids

## Abstract

Emulsifiers play a diverse role in the livestock industry. As a feed additive, it aids the digestion and absorption of added fat in feed to increase the productivity of broilers. The study aimed to investigate the effect of nutrient density levels and emulsifier supplementation on apparent total tract digestibility (ATTD) and apparent ileal digestibility (AID) in broilers. A total of 60 14-days- old Ross broiler chickens were randomly allocated into 4 group with 5 replicates and 3 chicks per cage for 14 days (phase 1 days 1 to 7 and phase 2 days 8 to 14). ATTD and AID were evaluated on days 7 and 14 using indigestible indicator method. Dietary treatment included: TRT1, Low nutrient diet without emulsifier; TRT2, Low nutrient diet + 0.05% emulsifier; TRT3, High nutrient diet without emulsifier; TRT4, High nutrient diet + 0.05% emulsifier. Results showed that the nutrient density diets have no effect (P>0.05) on ATTD and AID of amino acids. However, ATTD of dry matter, nitrogen, gross energy (GE) and crude protein and essential amino acid of lysine, valine, tryptophan and total essential amino acid and non-essential amino acid of proline and alanine, AID of dry matter and GE and essential amino acid of histidine and tryptophan and non- essential amino acid of proline and alanine were significantly increase (P <0.05) in chickens fed the emulsifier compared with the nutrient density. Therefore, high nutrient density diets emulsifier supplementation had a beneficial effect on ATTD and AID of amino acids.

## Introduction

Reducing production costs while maintaining product quality is a long-term goal in the poultry industry. Increasing the nutrient consumption of feed makes poultry production more efficient and sustainable (Maharjan et al., 2021). A major benefit of emulsifier supplements is often linked to improved fat digestion (Upadhaya et al., 2018). Consequently, exogenous emulsifiers can be used as feed additives to increase feed energy utilization and sustain high production in broiler chickens.

Emulsifiers, such as hydrophilic and hydrophobic groups, have been shown in many studies to increase amino acid quality by improving fat absorption and digestion, as a result, they are now a common ingredient in livestock and poultry feed (Ko et al., 2023; Sun and Kim, 2019). Various studies have shown inconsistent results regarding the significant impact of emulsifiers on feed digestibility when added directly to poultry diets (Liu et al., 2020; Shen et al., 2021). Roy et al. (2010) reported that the broiler digestion is unstable and their small digestive tracts make it difficult for external emulsifiers to improve fat digestibility. This enzyme activity increases the absorption of dietary fats and increases the utilization of ingested GE by breaking down large fat globules into smaller lipid droplets and promoting the incorporation of free fatty acids into micelles (Zurak et al., 2024). To increase the effectiveness of emulsifiers in the food industry, emulsifiers in food can often be consumed as emulsified fats (Zeng et al., 2023). These data may suggest that intestinal amphipathic external emulsifiers interact with proteins and hydrophobic amino acids in the diet. On the other hand, little is known about how emulsifiers and proteins, or amino acids interact with the digestive system. Therefore, the objective of this study was to evaluate the effect of low and high nutrient density and emulsifier supplementation on apparent total tract digestibility (ATTD) and apparent ileal digestibility (AID) in broilers.

## Materials and methods

All animal procedures used in this experiment were reviewed and approved by the Institutional Animal Care and Use Committee of Dankook University (Cheonan, Republic of Korea). (DK-1-2319)

### Experimental design, animals, and diets

A total of 60 14-day-old Ross 308 broiler chicks were selected in this study and raised in stainless steel cages (0.57 × 1.14 m) and fed experimental feed for 14 days. The house was provided with proper lighting and ventilation. Feed and water were provided ad libitum and diets were prepared. Birds were randomly assigned to 4 treatments based on the initial body weight (277 ± 21.06g). There were 5 replicates per treatment and 3 birds per cage. Dietary treatment included: TRT1, Low nutrient diet without emulsifier; TRT2, Low nutrient diet + 0.05% emulsifier; TRT3, High nutrient diet without emulsifier; TRT4, High nutrient diet + 0.05% emulsifier. Experimental periods were divided into 2 phases (phase 1, d1–7; phase 2, d8–14). Diets were formulated according to the recommendation of NRC (1994) Table 1.

**Table 1:** Feed composition of broiler (as fed-basis).

Items	Low Density	High Density
Ingredients (%)		
Corn	72.28	64.82
Soybean meal (48%)	6.78	23.30
Corn gluten meal (60%)	16.80	5.78
Tallow	-	2.50
TCP	2.50	2.40
Limestone	0.44	0.41
Salt	0.36	0.36
Methionine (99%)	0.05	0.10
Lysine	0.59	0.13
Mineral mix^1^	0.10	0.10
Vitamin mix^2^	0.10	0.10
Total	100.00	100.00
Calculated value		
Crude protein, %	20.00	20.00
Ca, %	1.00	1.00
P, %	0.80	0.80
Available P, %	0.57	0.57
Lys, %	1.00	1.00
Met, %	0.45	0.45
Metabolizable energy, kcal/kg	3250	3300
FAT,%	3.41	5.33
Fiber,%	2.11	2.45
Ash,%	4.97	5.60

1 Provided per kg of complete diet: 37.5 mg Zn (as ZnSO_4_); 37.5 mg Mn (as MnO_2_); 37.5 mg Fe (as FeSO_4_·7H_2_O); 3.75 mg Cu (as CuSO_4_·5H_2_O); 0.83 mg I (as KI); and 0.23 mg Se (as Na_2_SeO_3_·5H_2_O).

2 Provided per kg of complete diet: 15,000 IU of vitamin A, 3,750 IU of vitamin D_3_, 37.5 IU of vitamin E, 2.55 mg of vitamin K_3_, 3 mg of Thiamin, 7.5 mg of Rivoflavin, 4.5 mg of vitamin B_6_, 24 ug of vitamin B_12_, 51 mg of Niacin, 1.5 mg of Folic acid, 0.2 mg of Biotin and 13.5 mg of Ca-Pantothenate. TCP, Total Crude Protein;

### Experimental and laboratory analysis

Between days 1 and 14, broiler chicks were provided with a diet containing 0.2% chromium oxide. To assess the apparent total tract digestibility of nutrients, fecal samples were collected from day 5 to day 7 and from day 12 to day 14 from each pen. Special care was taken to ensure that the collected fecal samples were devoid of any feathers or residual feed. After collection, the fecal samples were mixed together, dried in an oven at 65°C for 72 hours, and then ground into a fine powder to pass through a 1-mm sieve. Analysis of dry matter (DM) and nitrogen (N) content in both feed and fecal samples was carried out following the methods outlined by AOAC International in 2022. The gross energy (GE) was determined using an automatic adiabatic oxygen bomb calorimeter (Parr 6300 Calorimeter, Moline, IL). Chromium concentration was measured using UV absorption spectrophotometry (UV-1201, Shimadzu, Kyoto, Japan). The equation for calculating digestibility was as follows: ATTD (%) = (1−((Nf × Cd)/ (Nd × Cf))) × 100, where Nf = nutrient concentration in feces (% DM), Nd = nutrient concentration in diet (% DM), Cf = chromium concentration in feces (% DM), and Cd = chromium concentration in diet (% DM).

On the final day of the 2 phases (day7, day14), ileal digesta samples were collected from a portion of the small intestine located between Meckel's diverticulum and the ileocecal junction to assess the apparent ileal digestibility (AID) of nutrients. The digesta samples were analyzed for crude protein (nitrogen × 6.25) and dry matter content following the procedures established by AOAC (2007). Chromium concentration was determined using UV absorption spectrophotometry (UV-1201, Shimadzu, Kyoto, Japan) based on the methods described by Williams et al. (1962). Nitrogen content was measured using a Kjeltec 2300 Analyzer (Foss Tecator AB, Hoeganaes, Sweden). GE was determined using a Parr 6100 oxygen bomb calorimeter (Parr Instrument Co., Moline, IL, USA). The individual amino acid composition in the digesta samples was analyzed using an amino acid analyzer (Beckman 6300; Beckman Coulter, Inc., Fullerton, CA, USA) and high-performance liquid chromatography (HPLC) following acid hydrolysis for 24 hours in 6 N–HCl at 110°C and vaporization at 50°C in an evaporator (HAHNSHIN S&T Co. Ltd. Kyunki-do, South Korea). The apparent ileal digestibility of amino acids (AID) was calculated according to the method proposed by Hossain et al. (2016). The apparent ileal amino acid (AA) digestibility was determined using the formula proposed by Wu et al. (2020): AID (%) = 100 − [100 * (AA digesta * Cr_2_O_3_ diet)/(AA diet * Cr_2_O_3_ digesta)], where AA diet and AA digesta represent the concentrations (mg/kg DM) of amino acids in the diet and digesta, respectively, and Cr_2_O_3_diet and Cr_2_O_3_ digesta denote the concentrations (mg/kg DM) of the indigestible marker (chromium oxide) in the diet and digesta, respectively. To standardize the apparent ileal AA digestibility, the average values for basal endogenous AA losses were calculated using the formula proposed by Muniyappan et al. (2022): AAEL (g/kg) = AA digesta * (Cr_2_O_3_diet / Cr_2_O_3_ digesta), where AAEL represents the average endogenous AA loss in the casein diet (g/kg of DM).

### Statistical analyses

Data were analyzed as a completely randomized design with 2 × 2 factorial arrangements using GLM procedures (SAS, 1996) when significant differences were obtained. Variability in the data was expressed as the standard error (SE) and significance was set at p< 0.05.

## Results

### Apparent total tract digestibility of amino acids

The effect of high and low nutrient diet with emulsifiers supplemented on the apparent total tract digestibility of amino acids is shown in Table 2. The supplementation of emulsifier with high nutrient diet had a significantly increased (p<0.05) on ATTD of dry matter, nitrogen, GE and crude protein and essential amino acid of lysine and total essential amino acid and non-essential amino acid of proline and alanine and essential amino acid of valine, tryptophan and total essential amino acid and nonessential amino acid of proline and alanine at week 1 and 2 compared to low nutrient diet with emulsifiers supplemented. There were no significant effects (p>0.05) of nutrient density and interaction effects.

**Table 2:** The effect of high and low nutrient diet with emulsifier supplementation on apparent total tract digestibility in broilers^1^.

Items, %	Low nutrient density		High nutrient density		SEM^2^	*P*-value^3^		
	0% (TRT1)	0.05% (TRT2)	0% (TRT3)	0.05% (TRT4)		Nutrient effect	Emulsifier effect	Interaction
**Apparent Total Tract Digestibility**								
**Week 1**								
Dry matter	64.60	66.56	65.68	68.67	1.05	0.1499	0.0321	0.6311
Nitrogen	62.26	64.96	64.04	66.98	0.92	0.0546	0.0073	0.8982
Energy	63.24	65.63	64.67	67.12	1.08	0.1960	0.0399	0.9724
**Week 2**								
Dry matter	68.57	71.54	70.92	73.70	1.78	0.2238	0.1262	0.9586
Nitrogen	66.70	69.69	68.93	71.84	1.76	0.2301	0.1123	0.9839
Energy	67.32	70.43	69.91	72.22	1.72	0.2215	0.1347	0.8191
**Week 1**								
Crude protein	66.87	69.54	68.77	70.96	1.13	0.1616	0.0480	0.8337
Crude fat	66.54	69.38	68.57	70.71	1.20	0.1815	0.0556	0.7738
Phosphorus	61.81	62.59	62.22	63.08	1.41	0.7572	0.5695	0.9778
**Essential amino acid**								
Threonine	71.28	72.69	71.89	74.84	1.33	0.3140	0.1202	0.5684
Valine	74.58	76.25	75.93	78.39	1.02	0.1069	0.0605	0.7050
Isoleucine	75.67	78.10	77.37	79.55	1.33	0.2530	0.1019	0.9262
Leucine	78.72	79.48	79.16	80.17	1.07	0.6040	0.4234	0.9080
Phenylalanine	78.93	79.76	79.27	80.64	1.09	0.5818	0.3270	0.8091
Histidine	76.91	78.51	77.99	79.90	0.89	0.1840	0.0671	0.8625
Lysine	80.21	82.35	81.24	83.33	0.99	0.3258	0.0482	0.9762
Arginine	81.52	82.63	82.07	83.45	0.82	0.4158	0.1500	0.8720
Methionine	85.36	86.33	86.06	87.08	1.65	0.6652	0.5556	0.9886
Tryptophan	78.44	81.29	80.33	82.83	1.29	0.2021	0.0544	0.8912
TEAA	78.16	79.74	79.13	81.02	0.70	0.1280	0.0255	0.8271
**Non-Essential amino acid**								
Aspartic acid	75.04	76.43	75.61	76.91	1.62	0.7505	0.4174	0.9787
Serine	76.39	77.51	76.80	78.10	1.47	0.7399	0.4245	0.9521
Glutamic acid	70.99	72.05	71.55	72.68	1.01	0.5643	0.2951	0.9735
Proline	74.49	76.34	75.58	77.82	0.86	0.1570	0.0308	0.8271
Glycine	76.70	77.71	77.28	78.30	1.28	0.6542	0.4384	0.9957
Alanine	73.65	76.22	75.65	77.29	0.82	0.0793	0.0206	0.5781
Tyrosine	77.32	78.31	77.85	78.89	0.87	0.5313	0.2605	0.9739
Cystine	69.67	70.24	69.98	70.69	1.37	0.7838	0.6464	0.9587
TNEAA	74.28	75.60	75.04	76.34	0.88	0.4096	0.1568	0.9902
Total Amino Acid	76.44	77.90	77.31	78.94	0.74	0.2114	0.0526	0.9128
**Week 2**								
Crude protein	69.99	72.95	72.00	74.13	1.29	0.2344	0.0661	0.7517
Crude fat	70.16	72.80	71.98	74.43	1.27	0.1925	0.0628	0.9389
Phosphorus	64.95	66.24	65.56	66.76	1.85	0.7667	0.5111	0.9809
**Essential amino acid**								
Threonine	74.01	77.09	76.34	78.60	1.43	0.1977	0.0800	0.7761
Valine	77.96	80.84	80.59	82.24	0.97	0.0555	0.0338	0.5388
Isoleucine	78.84	81.92	81.47	83.08	1.11	0.1065	0.0507	0.5173
Leucine	82.35	83.11	82.76	83.71	0.82	0.5440	0.3128	0.9139
Phenylalanine	82.79	83.63	83.14	84.34	1.25	0.6776	0.4274	0.8858
Histidine	80.28	82.45	82.08	84.03	1.01	0.1113	0.0577	0.9144
Lysine	83.60	85.81	85.06	87.47	1.38	0.2769	0.1138	0.9426
Arginine	85.22	86.29	85.61	87.15	1.03	0.5531	0.2222	0.8226
Methionine	89.65	90.64	90.21	91.43	1.73	0.7026	0.5326	0.9475
Tryptophan	82.39	85.08	83.85	86.63	1.05	0.1734	0.0195	0.9680
TEAA	81.71	83.69	83.11	84.87	0.81	0.1312	0.0352	0.8929
**Non-Essential amino acid**								
Aspartic acid	79.08	79.82	79.58	80.33	1.27	0.6941	0.5675	0.9981
Serine	81.48	82.00	81.93	82.39	1.15	0.7218	0.6767	0.9796
Glutamic acid	74.77	75.79	75.28	76.10	1.05	0.6996	0.3939	0.9232
Proline	77.51	79.99	79.22	80.96	0.99	0.1950	0.0487	0.7094
Glycine	80.41	81.28	80.92	81.99	0.92	0.5126	0.3065	0.9135
Alanine	77.26	80.26	79.50	81.43	1.03	0.1184	0.0302	0.6142
Tyrosine	80.39	81.54	80.83	82.21	1.03	0.5996	0.2402	0.9136
Cystine	72.57	73.32	72.94	74.01	1.41	0.7121	0.5268	0.9093
TNEAA	77.93	79.25	78.78	79.93	0.84	0.3769	0.1603	0.9223
Total Amino Acid	80.03	81.71	81.18	82.67	0.79	0.2020	0.0630	0.9013

1 Abbreviation: TRT1, Low nutrient diet; TRT2, Low nutrient diet + emulsifier 0.05%; TRT3, High nutrient diet; TRT4, High nutrient diet + emulsifier 0.05%^.^; TEAA- Total Essential Amino Acid; TNEAA, Total Non-Essential Amino Acid

2 Standard error of means

3 Means in the same row with different superscript differ significantly (P < 0.05).

### Apparent ileal digestibility of amino acids

There were no significant effects (p> 0.05) of nutrient density diet on the apparent ileal digestibility of amino acids of boilers as shown in Table 3. Emulsifier supplementation improved (p<0.05) AID of dry matter and GE during week 2. In addition, emulsifier supplementation had a significantly increase (p<0.05) essential amino acid of histidine and tryptophan and non- essential amino acid of proline and alanine at week 1 and essential amino acid of valine, tryptophan and total essential amino acid and non- essential amino acid of proline and alanine at week 2. No interaction effects were observed between diet density and emulsifiers for the apparent ileal digestibility of amino acids.

**Table 3:** The effect of high and low nutrient diet with emulsifier supplementation on apparent ileal digestibility in broilers^1^.

Items, %	Low nutrient density		High nutrient density		SEM^2^	*P*-value^3^		
	0% (TRT1)	0.05% (TRT2)	0% (TRT3)	0.05% (TRT4)		Nutrient effect	Emulsifier effect	Interaction
**Apparent Ileal Digestibility**								
**Week 1**								
Dry matter	62.36	64.07	63.13	66.44	1.51	0.3135	0.1161	0.6064
Nitrogen	60.24	62.24	61.44	64.76	1.48	0.2262	0.0917	0.6621
Energy	61.51	63.76	62.87	65.81	1.57	0.2938	0.1169	0.8285
**Week 2**								
Dry matter	66.36	69.18	67.91	71.51	1.45	0.1997	0.0420	0.7948
Nitrogen	64.32	67.12	66.19	69.78	1.60	0.1757	0.0626	0.8093
Energy	65.47	68.70	67.52	70.92	1.42	0.1520	0.0328	0.9529
**Week 1**								
Crude protein	65.06	67.60	66.73	69.07	1.52	0.3177	0.1270	0.9467
Crude fat	63.95	66.76	65.70	68.01	1.55	0.3459	0.1179	0.8726
Phosphorus	59.74	60.92	60.30	61.31	2.06	0.8191	0.6031	0.9676
**Essential amino acid**								
Threonine	69.37	70.88	70.09	72.57	1.28	0.3583	0.1374	0.7047
Valine	72.86	75.19	74.38	76.67	1.18	0.2246	0.0686	0.9881
Isoleucine	73.36	76.25	75.94	77.30	1.11	0.1228	0.0750	0.4999
Leucine	76.86	77.45	77.14	78.09	1.05	0.6668	0.4724	0.8659
Phenylalanine	76.62	78.14	77.68	78.98	1.46	0.5245	0.3506	0.9398
Histidine	74.33	76.81	76.12	77.88	0.91	0.1341	0.0326	0.7002
Lysine	78.20	80.30	79.62	81.34	1.10	0.2803	0.1015	0.8629
Arginine	80.00	81.10	80.57	81.44	0.90	0.6189	0.2912	0.8996
Methionine	84.08	84.90	84.52	85.67	1.81	0.7426	0.5929	0.9254
Tryptophan	76.03	79.53	78.34	80.92	1.26	0.1606	0.0279	0.7211
TEAA	76.17	78.05	77.44	79.09	0.85	0.1956	0.0545	0.8905
**Non-Essential amino acid**								
Aspartic acid	73.63	74.40	74.21	75.03	1.45	0.6816	0.5905	0.9886
Serine	75.10	76.09	75.30	76.43	1.32	0.8430	0.4321	0.9577
Glutamic acid	69.24	70.26	69.79	70.80	1.29	0.6773	0.4434	0.9964
Proline	72.62	74.35	73.82	75.95	0.81	0.1041	0.0305	0.8153
Glycine	75.13	75.80	75.48	76.57	1.11	0.6181	0.4377	0.8476
Alanine	71.76	73.53	72.92	75.41	0.95	0.1275	0.0394	0.7097
Tyrosine	74.54	75.89	75.22	76.63	1.22	0.5701	0.2757	0.9820
Cystine	66.62	68.00	67.34	68.41	1.66	0.7378	0.4726	0.9270
TNEAA	72.33	73.54	73.01	74.40	0.96	0.4331	0.1945	0.9248
Total Amino Acid	74.46	76.05	75.47	77.00	0.88	0.2805	0.0959	0.9777
**Week 2**								
Crude protein	68.10	70.76	69.83	72.64	1.38	0.2086	0.0644	0.9566
Crude fat	69.10	71.22	70.57	72.98	1.30	0.2314	0.1014	0.9128
Phosphorus	63.48	64.96	64.32	65.42	1.88	0.7336	0.5033	0.9203
Essential amino acid								
Threonine	72.56	75.47	74.72	77.15	1.50	0.2170	0.0931	0.8724
Valine	76.59	79.31	78.99	80.81	1.03	0.0764	0.0422	0.6681
Isoleucine	77.25	80.39	79.96	81.63	1.16	0.1090	0.0550	0.5380
Leucine	80.74	81.57	80.91	82.34	0.88	0.5979	0.2199	0.7375
Phenylalanine	81.20	82.27	81.79	82.78	1.28	0.6737	0.4357	0.9749
Histidine	78.87	80.94	80.58	82.69	1.06	0.1235	0.0668	0.9845
Lysine	82.10	84.42	83.67	85.92	1.42	0.2941	0.1268	0.9778
Arginine	83.90	84.75	84.08	85.78	1.07	0.5788	0.2506	0.6968
Methionine	88.19	89.13	88.71	90.09	1.76	0.6816	0.5197	0.9018
Tryptophan	80.80	83.49	82.44	85.19	1.05	0.1300	0.0196	0.9776
TEAA	80.22	82.17	81.59	83.44	0.88	0.1556	0.0467	0.9547
**Non-Essential amino acid**								
Aspartic acid	77.43	78.28	77.95	78.91	1.32	0.6683	0.5047	0.9672
Serine	79.90	80.67	80.50	80.76	1.17	0.7704	0.6639	0.8302
Glutamic acid	73.22	74.28	73.78	74.76	1.12	0.6478	0.3765	0.9719
Proline	75.99	78.69	77.96	79.59	1.00	0.1711	0.0458	0.6005
Glycine	78.99	79.76	79.53	80.47	0.98	0.5325	0.3953	0.9269
Alanine	75.81	78.81	78.07	79.98	1.06	0.1260	0.0345	0.6158
Tyrosine	78.72	79.94	79.78	80.56	1.12	0.4648	0.3828	0.8463
Cystine	71.01	71.88	71.52	72.59	1.49	0.6896	0.5262	0.9490
TNEAA	76.39	77.79	77.39	78.45	0.92	0.3769	0.1950	0.8550
Total Amino Acid	78.51	80.22	79.72	81.22	0.87	0.2242	0.0836	0.9083

1 Abbreviation: TRT1, Low nutrient diet; TRT2, Low nutrient diet + emulsifier 0.05%; TRT3, High nutrient diet; TRT4, High nutrient diet + emulsifier 0.05%; TEAA- Total Essential Amino Acid; TNEAA, Total Non-Essential Amino Acid

3 Means in the same row with different superscript differ significantly (P < 0.05).

## Discussion

In the broiler industry, production costs can be saved by using nutrient-dense diets, modifying nutrients, and adding emulsifiers. Therefore, this study was conducted to evaluate the effect of emulsifier supplementation of low- and high-nutrient-dense diets on ATTD, AID and amino acids. A realistic way to increase production in the broiler industry is to modify the nutrient density of the diet. In addition, it is essential to determine the appropriate nutrient density level before adding emulsifier additives to alter the matrix value of the emulsifier. The present study showed that the nutrient density diet had did not effects on the ATTD, AID and amino acids. This study is consistent with findings from previous research that fed nutrient density diets which resulted in no effect on ATTD (Zeng et al., 2023). On the other hand, Ko et al. (2023) reported that the dietary inclusion of nutrient density diets had increased AID of dry matter, nitrogen and amino acid of chickens.

In the current study, birds fed high nutrient diet with emulsifier supplementation had a significant enhancement on ATTD of dry matter, nitrogen, GE and crude protein, essential amino acid of lysine, valine, tryptophan and total essential amino acid and non-essential amino acid of proline and alanine compared to low nutrient diet with emulsifier supplementation. In addition, AID of dry matter and GE and essential amino acid of histidine and tryptophan and non- essential amino acid of proline and alanine had significant improvement on high nutrient diet with emulsifier supplementation. A higher percentage of amino acids from endogenous sources results in increase amino acid concentrations in digesta and consequently lower amino acid intakes (Zhao and Kim, 2017). Exogenous emulsifier improves the active surface of fat in ingested feed particles and increases the ability of enzymes to reach the particles (Gholami et al., 2024). Such changes may improve the digestibility of certain nutrients, including DM and CP (Liu et al., 2020; Zhao and Kim, 2017). Zampiga et al. (2016) reported that the dietary inclusion emulsifier could increase ATTD of dry matter and nitrogen in chickens. In our investigation, the emulsifier-loaded group showed improved CP digestibility and better hydrophobic amino acid digestibility, including branched-chain amino acids. Furthermore, the study revealed significant interaction effects on the digestibility of amino acids, suggesting that the emulsifier added to a low-nutrient-dense diet had a greater effect on improving digestibility than a nutrient-dense diet. Nutrient digestibility results of the present study indicate that protein, rather than fat, is the primary driver of nutrient utilization for growth in the broiler maintenance and emulsifier group. This study adds to the body of research showing that emulsifier supplementation can counteract the effects of nutrient density by improving the efficiency of nutrient and amino digestion. It also suggested that emulsifier supplementation affects metabolism energy by regulating protein and amino acid utilization, particularly in nutrient-density diets. In every parameter tested, there was no correlation between different levels of emulsifier and nutrient density. Consequently, no additive effects were noted. In conclusion, the nutrient density diets were found to have no influence on chickens ATTD and AID of amino acids. However, the addition of emulsifiers improved the ATTD and AID of amino acids, while counteracting growth retardation caused by nutrient-dense diets. Also, research needs to be done on how emulsifiers affect aspects of intestinal protein and amino acid digestion.

## Declaration of competing interest

This study has no potential conflict of intertest.
